# Immunotherapy for the prevention of high-risk oral disorders malignant transformation: the IMPEDE trial

**DOI:** 10.1186/s12885-021-08297-3

**Published:** 2021-05-17

**Authors:** Cristina Gurizzan, Luigi Lorini, Alberto Paderno, Michele Tomasoni, Gabriele Zigliani, Anna Bozzola, Laura Ardighieri, Simonetta Battocchio, Eliana Bignotti, Antonella Ravaggi, Chiara Romani, Loris De Cecco, Mara Serena Serafini, Rosalba Miceli, Elena Bardellini, Alessandra Majorana, Cesare Piazza, Paolo Bossi

**Affiliations:** 1 Medical Oncology Unit, Department of Medical and Surgical Specialities, Radiological Sciences and Public Health University of Brescia, ASST-Spedali Civili, Piazzale Spedali Civili 1, 25123 Brescia, Italy; 2 Unit of Otorhinolaryngology – Head and Neck Surgery, Department of Medical and Surgical Specialities, Radiological Sciences and Public Health University of Brescia, ASST-Spedali Civili, Brescia, Italy; 3 Unit of Pathology, Department of Molecular and Translational Medicine, University of Brescia, ASST-Spedali Civili, Brescia, Italy; 4 ‘Angelo Nocivelli’ Institute of Molecular Medicine, University of Brescia and ASST-Spedali Civili, Brescia, Italy; 5 Department of Applied Research and Technology Development, Integrated Biology Platform, Fondazione IRCCS Istituto Nazionale dei Tumori di Milano, Milan, Italy; 6 Department of Applied Research and Technical Development, Medical Statistics and Biometry Unit, Fondazione IRCCS Istituto Nazionale dei Tumori, Milan, Italy; 7 Dental Clinic, Oral Medicine Unit - Department of Medical and Surgical Specialties, Radiological Science and Public Health University of Brescia, ASST-Spedali Civili, Brescia, Italy

**Keywords:** Oral potentially malignant disorder, LOH, Malignant transformation, Immunotherapy, Prevention, Oral cancer

## Abstract

**Background:**

Oral Potentially Malignant Disorders (OPMD) have a non-negligible malignant transformation rate of up to 8%. Loss of heterozygosity (LOH) in critical chromosomal loci has proven to be the most effective marker in defining the risk of transformation and it is found in about 28% of OPMD and may therefore identify patients carrying higher risk. To date, clinical management of OPMD is limited to surgical excision and clinical surveillance, which however do not fully prevent oral cancer development. Immune system has been shown to play a key role in transformation surveillance mechanism and an immunosuppressive imbalance may be responsible for progression to cancer. Given all these considerations, we designed a clinical trial with the aim to prevent OPMD neoplastic transformation and revert the LOH status.

**Methods:**

This is a phase II, open label, single arm, multicentric trial involving Italian referral centres and expected to enrol 80 patients out of a total of 175 screened. Patients who meet all inclusion criteria and test positive for LOH after an incisional biopsy of the OPMD will undergo a short course of immunotherapy with 4 administration of avelumab. After 6 months since treatment start, resection of the entire OPMD will be performed and LOH assessment will be repeated. The follow-up for malignant transformation and safety assessment will last 30 months from the end of treatment, for a total planned study duration of approximately 5.5 years.

**Discussion:**

Restoring the activity of immune system through checkpoint inhibitor may play a crucial role against malignant transformation of OPMD by reverting the balance in favour of immune control and preventing cancer occurrence.

**Trial registration:**

This trial was prospectively registered in ClinicalTrials.gov as NCT04504552 on 7th August 2020.

**Supplementary Information:**

The online version contains supplementary material available at 10.1186/s12885-021-08297-3.

## Background

Oral Potentially Malignant Disorders (OPMD) represent the most common oral precancerous condition, with an estimated prevalence rate worldwide of 4.5% [1] and, according to a recent meta-analysis, an overall potential of neoplastic transformation of 7.9% [2].

Among molecular markers, the most effective in predicting the risk of progression to oral cancer is loss of heterozygosity (LOH), which may appear in tissues regardless of their histological grade of dysplasia [3, 4]. This chromosomal profile may be found in about 28% of OPMD [5]. Patients carrying OPMD with LOH at 3p14 and/or 9p21 plus LOH at another locus have an expected 3-year risk of developing oral cancer of 35%. Moreover, when a patient previously suffered from oral cancer, the 3-year risk of malignant transformation for OPMD with defined LOH reaches 69% [6]. The presence of tumour suppressor genes at these chromosomal loci explains the potential for cancer development in presence of such alterations.

The clinical management of small OPMD is excision by electrocautery or laser. However, treatment is not always effective in preventing oral cancer. Lesions recur frequently and may subsequently progress, with tumours developing in the same or adjacent anatomical regions [7]. For larger OPMD, clinical management may be limited to lifelong surveillance as an alternative to potentially morbid repeated, multi-step surgical treatments. To improve clinical care of OPMDs, the arsenal of treatments should be expanded.

OPMD may be considered as the equilibrium phase of the immunoediting concept, i.e. a dynamic process between the tumour cells and the immune system including immunosurveillance or tumour progression [8, 9]. Thus, an imbalance in the immunosuppressive microenvironment is a possible key for malignant transformation. In this regard, the activation of the PD-1/PD-L1 pathway has a central role, witnessed by the expression of PD-L1 by multiple cell types within the OPMD microenvironment (tumour-associated macrophages, fibroblasts, lymphocytes), and by the fact that PD-L1 expression in epithelial and subepithelial cells is associated with malignant transformation [10]. The use of checkpoint inhibitors in this setting is justified by this rationale.

Employing intermediate end-point markers during preventive strategies against OPMD may allow the conduction of smaller trials, able to give insights for designing larger ones and to better select the population receiving benefit from a given treatment. In this regard, the evaluation of phenotypic changes (reduction in size or in grade of dysplasia) may not be enough to assess the potential benefit of an intervention. Modulation of molecular markers may be in fact a more precise indicator of oral cancer risk in patients with OPMD. Thus, the change in LOH in critical loci may be considered an intermediate end-point biomarker of prevention, as well as a predictor of cancer risk at baseline. Previous experience with anti-EGFR agent showed the feasibility of such measures in a prevention trial [5].

## Methods and design

### Objectives

#### Primary objective

The primary objective is to revert the risk of malignant transformation of OPMD through a short course of immunotherapy with avelumab, a *PD-L1*-blocking monoclonal antibody agent. This objective will be evaluated both with an intermediate endpoint (restore of LOH status) and in its final endpoint (malignant transformation).

#### Secondary objectives

- To evaluate the safety of an immunotherapeutic approach in OPMD
- To assess the change of histological grade of OPMD
- To discover genomic predictive tools able to foresee response to avelumab

### Endpoints

#### Co-primary endpoints

- Recurrence or malignancy-free survival since the start of immunotherapy, for which the events of interest are the recurrence of OPMD with LOH or malignant transformation
- Change in LOH status (positive to negative) of OPMD after 6 months since the start of avelumab. This is defined as the disappearance of any high-risk LOH (and the non-appearance of any other high-risk LOH) in the histological exam of the OPMD after immunotherapy treatment

#### Secondary endpoints

- Grade 3–5 adverse events or any treatment interruption due to toxicities (safety)
- Change of histological grading of OPMD
- Identification of multi-omics signatures associated with response to immunotherapy

### Patient population and eligibility criteria

#### Inclusion criteria

1. Signed written informed consent;
2. Male or female > 18 years of age;
3. ECOG Performance status (PS) 0–1;
4. Clinical and histological evidence of OPMD without prior history of oral cancer and with high risk of malignant transformation as defined by LOH at 3p14 and/or 9p21 plus at least in one additional chromosomal site (4q, 8p,11q,13q, or 17p), or patients with a prior oral cancer history and LOH at 3p14 and/or 9p21 (LOH defined according to EPOC trial [5]). These conditions define the “LOH positivity”;
5. OPMD with a histological definition of dysplasia and a diameter of at least 20 mm;
6. Willing to provide tissue from newly obtained oral biopsies;
7. Not receiving chronic systemic steroidal therapy or any immunosuppressive therapy within 7 days prior to the first treatment;
8. Absence of active autoimmune disease that required systemic treatment in the past 2 years;
9. Adequate bone marrow function: neutrophils > 1.5 × 10^9^/L, platelets > 100 × 10^9^/L, haemoglobin > 9 g/dL;
10. Adequate liver function: bilirubin < 2 times above the upper normal limit (except known medical reasons not interfering with liver function, such as Gilbert disease), AST, ALT, ALP, GGT < 3 times above the upper normal limits;
11. Adequate renal function: calculated or analysed creatinine clearance > 60 mL/min;
12. If of childbearing potential, willingness to use effective contraceptive method (Pearl Index < 1; e.g. oral contraceptive [pill], hormone spiral, hormone implant, transdermal patch, a combination of two barrier methods [condom and diaphragm], sterilization, sexual abstinence) for the study duration and 2 months post-dosing.

#### Exclusion criteria

1. Previous immunotherapy (anti-PD-1, anti-PD-L1, anti-PD-L2, anti-CTLA-4 antibody or any other antibody or drug specifically targeting T-cell co-stimulation or immune checkpoint receptors);
2. Biopsy-proven oral lichen planus;
3. Diagnosis of prior immunodeficiency or organ transplant requiring immunosuppressive therapy, or known human immunodeficiency virus (HIV) or acquired immunodeficiency syndrome (AIDS)-related illness;
4. Any test for hepatitis B virus (HBV) or hepatitis C virus (HCV) indicating acute or chronic infection;
5. Vaccination within 4 weeks of the first dose of avelumab and while on trial is prohibited except for the administration of inactivated vaccines (for example, inactivated influenza vaccines);
6. Clinically significant cardiovascular disease, e.g. cardiac failure of New York Heart Association classes III-IV, uncontrolled coronary artery disease, cardiomyopathy, uncontrolled arrhythmia, uncontrolled hypertension, or history of myocardial infarction in the last 12 months;
7. Significant neurologic or psychiatric disorders including dementia or seizures;
8. Active uncontrolled infection (requiring IV antibiotics) or active tuberculosis;
9. Active disseminated intravascular coagulation;
10. Other serious underlying medical conditions which could impair the ability of the patient to participate in the study;
11. Having participated in another clinical trial or having received any investigational agent in the preceding 30 days before study entry;
12. Known allergic/hypersensitivity reaction to any of the components of the treatment;
13. Pregnancy (absence confirmed by serum/urine beta HCG) or breastfeeding;
14. Other active malignancy within 3 years, with the exception of a history of either one of the following, adequately treated: oral cancer, basal cell carcinoma of the skin, pre-invasive carcinoma of the cervix, superficial bladder cancer, carcinoma in situ of the prostate, cervix or breast;
15. Legal incapacity or limited legal capacity;
16. Medical, psychological or socio-geographical conditions or situations which, in the opinion of the investigator, would not permit the patient to complete the study or sign meaningful informed consent;
17. Active, known or suspected autoimmune diseases. Patients with vitiligo, type I diabetes mellitus, residual hypothyroidism due to autoimmune condition only requiring hormone replacement, psoriasis not requiring systemic treatment, or conditions not expected to recur in the absence of an external trigger are permitted to be enrolled;
18. Conditions requiring systemic treatment with either corticosteroids (> 10 mg daily prednisone equivalents) or other immunosuppressive medications within 7 days of study drug administration. Inhaled or topical steroids and adrenal replacement doses > 10 mg daily prednisone equivalents are permitted in the absence of active autoimmune disease.

### Study design

**Fig. 1:**
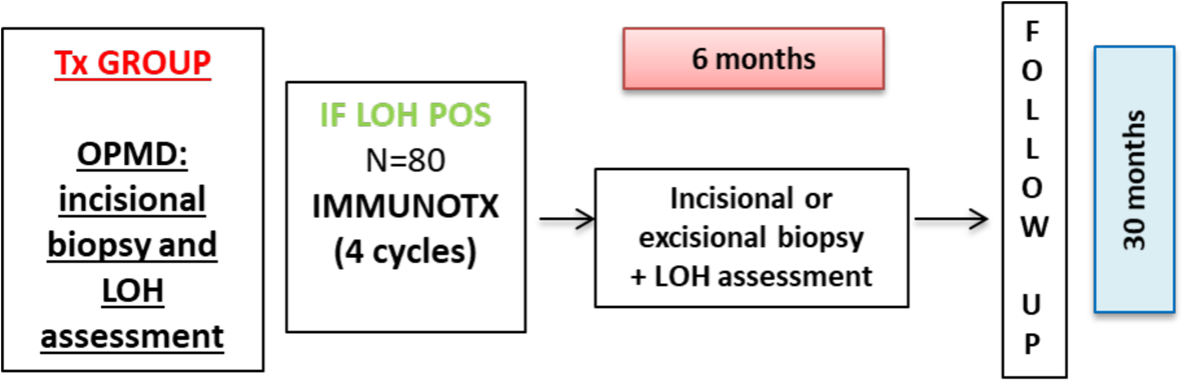
Study design

Study design is summarized in Fig. 1.

The first study phase of screening begins by establishing the subject’s initial eligibility and signing of the informed consent form (ICF). Patients who choose to participate in the study will then undergo an incisional biopsy of the OPMD and, only in case of dysplasia (regardless of grading), will be included in this study and test for LOH.

LOH negative subjects who meet all other inclusion criteria, will be treated as per clinical practice (e.g., OPMD surgical excision and/or follow-up). However, information on the malignancy free-survival period will be collected for these patients at 30 months from the end of the screening phase.

LOH positive subjects will then undergo a short course (4 cycles) of immunotherapy with avelumab flat dose (800 mg iv) every 2 weeks. Four administrations of the checkpoint inhibitor avelumab have been chosen with the rationale to induce a response in lesions whose immunological derangement is expected to be less severe than in advanced cancer [10]. According to the immunoediting concept, OPMD are considered in the equilibrium phase, but the discovery of high-risk LOH could represent the initial step of immunoescape; therefore, a short course of immunotherapy could be considered sufficient to reverse such a shift in immunocompetence. Moreover, a limited number of administrations could expose patients to less risk of adverse events. This period of treatment is also consistent with other preventive trials in OPMD, both with targeted agents (cetuximab for 2 months, according to Khan et al. [11]) and immunotherapy (pembrolizumab for 4 cycles, clinicaltrials.gov: NCT02882282).

After 6 months since treatment start, excisional biopsy of OPMD will be performed or, in case of not feasibility or excessive potential morbidity of a complete resection, another incisional biopsy will be performed and LOH assessment repeated. In case of complete clinical response, the site of previous OPMD will be biopsied, to define histology and to assess LOH even in presence of normal mucosa. The 6-month timeframe to assess changes in LOH has been chosen because it is relevant for the immune system to produce a shift in genetic markers and it is a period that may be consistent with clinical practice when re-assessing a lesion for surgery.

Subjects will be followed up with clinical examinations and safety assessment every month in the first year, then every 3 months for the following 18 months. Information about OPMD recurrence and malignancy-free survival will be collected.

The duration of the study from start of enrolment to data availability for final analysis of survival will be approximately 5.5 years.

The complete flowchart of study assessment and procedures is depicted in Additional File 1.

### Statistical methodology and planned analysis

#### Sample size determination

We consider that 28 and 69% of OPMD without and with history of previous oral cavity cancer, respectively, carry high-risk OPMD. We expect to enrol about 35% of the patients of the first group (*N* = 28) and 65% of the second group (*N* = 52) (overall sample size *N* = 80 patients). Therefore, the overall number of patients with OPMD to be screened is 175. As regards the first coprimary endpoint, a sample size of 80 to 85 patients will allow to reach an 80% power for testing differences of about 13% using a one-sided binomial test for one proportion at a significance level of 2.5% (Table 1).

**Table 1:** P0: probability of change in LOH status after 6 months of treatment with immunotherapy under the null hypothesis. P1: probability of change in LOH status after 6 months of treatment with immunotherapy under the alternative hypothesis

N	P0	P1	diff
80	0.20	0.34	0.14
80	0.17	0.30	0.13
83	0.16	0.29	0.13
85	0.19	0.32	0.13

As regards the second coprimary endpoint, a sample size of 78 to 83 patients will allow to reach a 80% power for testing differences of 20 to 22% (Table 2) using a one sample log-rank test at a significance level of 2.5% (2 years of accrual and 1 year of follow-up).

**Table 2:** S0: recurrence or malignancy-free survival probability at 3 years under the null hypothesis. S1: recurrence or malignancy-free survival probability at 3 years under the alternative hypothesis

N	S0	S1	diff
80	0.65	0.87	0.22
81	0.60	0.82	0.22
82	0.55	0.77	0.22
82	0.50	0.72	0.22
78	0.45	0.67	0.22
83	0.40	0.61	0.21
79	0.35	0.56	0.21
80	0.30	0.50	0.20

### Statistical analysis

The first coprimary endpoint is the recurrence or malignancy-free survival, for which the time is the interval from the date of the start of immunotherapy to the date of recurrence of OPMD with LOH or of malignant transformation or the date of death without recurrence/malignancy (if caused by treatment), whichever will occur first. Time will be censored at the date of last follow-up for patients alive and without events or dead for causes not related to the treatment. The recurrence or malignancy-free survival curve will be estimated with the Kaplan-Meier method.

The second co-primary endpoint is the change in LOH status (positive to negative) after 6 months of treatment with immunotherapy. The probability of change in LOH status after 6 months of treatment will be estimated with the corresponding relative frequency of patients with change among the total number of LOH positive patients at treatment start; the corresponding exact Clopper-Pearson 95% confidence interval will be estimated.

### Ethical considerations

#### Good clinical practice

This study is to be conducted according to globally accepted standards of good clinical practice (as defined in the ICH E6 Guideline for Good Clinical Practice, 1 May 1996), in agreement with the Declaration of Helsinki, in keeping with local regulations, and in compliance with the protocol. Personnel involved in conducting this study will be qualified by education, training, and experience to perform their respective tasks.

### Informed consent

A written informed consent will be obtained from all patients prior to study procedures start. Investigators must ensure that subjects are clearly and fully informed about the purpose, potential risks, and other critical issues regarding clinical studies in which they volunteer to participate. The sample informed consent form will adhere to the ethical principles that have their origin in the Declaration of Helsinki.

### Data confidentiality and personal data protection

Information about study subjects will be kept confidential and managed under the applicable laws and regulations. The data collection system for this study uses built-in security features to encrypt all data for transmission in both directions, preventing unauthorized access to confidential participant information.

## Discussion

Despite a major effort in identifying a chemopreventive strategy to reverse the process of neoplastic transformation, no pharmacological or natural agents have been proven to be effective so far. Several agents have been studied, including retinoids, cyclooxygenase-2 (COX2), EGFR inhibitors, p53-targeted agents, thiazolidinediones, and natural compounds such as antioxidants present in green tea extract [7].

The Erlotinib Prevention of Oral Cancer (EPOC) [5] trial randomized patients with OPMD and high-risk LOH profile (3p14 and/or 9p21 in case of history of oral cancer or LOH at 3p14 and/or 9p21 plus an additional chromosomal site if no history of oral cancer was collected) to receive either EGFR tyrosine kinase inhibitor erlotinib or placebo, in order to prevent oral cancer occurrence. No statistically significant differences were found in oral cancer-free survival (CFS) between the placebo and erlotinib arms. However, the 3-year oral CFS rate was higher in LOH-negative compared to LOH-positive group (87% vs. 74%), confirming the role of LOH as a biomarker of cancer risk.

We are waiting for data of the already closed (March 2018) phase 2 trial with vandetanib to evaluate efficacy on high-risk OPMD (NCT01414426).

Recently, several evidences are confirming the role of the immune system in premalignant lesions. Chaves and colleagues [8] demonstrated an increased CD8+ cells infiltrate in premalignant lesions not evolving in cancer in respect to those that preceded malignant transformation. Moreover, oral carcinoma was more infiltrated by CD8+ cells than its associated OPMD. In contrast, a significant increase in PD-L1 expression was shown in progressing compared to non-progressing oral dysplasias [9]. Patients suffering from oral leukoplakia and lower infiltration of TCD3+ cells seem to have a higher risk of malignant transformation, as well as those with high Th1 levels oral lesions [12]. In addition, a systemic inflammatory activity, with increased levels of alpha-TNF and pro-inflammatory cytokines both in plasma and saliva, has been detected in patients with squamous cell carcinoma of the head and neck and OPMD. However, a predictive impact on carcinogenic transformation risk has not yet been assessed based on the respective serum and salivary values [13].

Taken all together, these data confirm the importance of the inflammatory status of OPMD and pave the way to the study of checkpoint inhibitors to restore immune activity in order to prevent immune-escape and progression to oral cancer.

### Trial status

The IMPEDE study was prospectively registered in ClinicalTrials.gov as NCT04504552 on 7th August 2020. https://www.clinicaltrials.gov/NCT04504552

## Supplementary Information

**Additional file 1.** Flow Chart/Time and Events Schedule.

## Data Availability

The datasets used and/or analyzed during the current study will be available at the end of the study with the final report.

## Abbreviations

AE: Adverse event
ALP: Alkaline phosphatase
ALT: Alanine aminotransferase
AST: Aspartate aminotransferase
CTLA-4: Cytotoxic T-lymphocyte-associated antigen-4
ECG: Electrocardiogram
ECOG: Eastern Cooperative Oncology Group
GGT: Gamma-Glutamyl Transferase
HIV: Human immunodeficiency virus
ICF: Informed consent form
ICH: International Conference on Harmonization
LOH: Loss Of Heterozygosity
OPMD: Oral Potentially Malignant Disorders
PD-1: Programmed cell death 1
PD-L1: Programmed cell death ligand 1
PD-L2: Programmed cell death ligand 2
Q2W: Every 2 weeks
ULN: Upper limit of normal
PS: Performance status
