# Supplementary material for: Immunotherapy for the prevention of high-risk oral disorders malignant transformation: the IMPEDE trial
Source: BMC Cancer. 2021 May 17;21:561. doi: 10.1186/s12885-021-08297-3 (PMC8130439; doi:10.1186/s12885-021-08297-3)
Supplement: Supplementary file 1 — Additional file 1. Flow Chart/Time and Events Schedule. [file 12885_2021_8297_MOESM1_ESM.docx]

| **Period (duration)** | **Screening** (≤42 days) | **Baseline** (1 day) | **Treatment period**  (± 2 days – q2w until w7 and then as shown) | | | | | | | **Follow-up**  every month for the 1^st^ year, then every 3 months for 30 months |
| --- | --- | --- | --- | --- | --- | --- | --- | --- | --- | --- |
| **Visit** | **0** | **1** | **2** | **3** | **4** | **5** | **6** | **7** | **8** | 30/90 (±7days) from w24 for 30 months |
| **Day** | **-42 to 0** | **1** | **15** | **29** | **43** | **71** | **99** | **127** | **162** |  |
| **Week** | **-6 to 0** | **1** | **3** | **5** | **7** | **11** | **15** | **19** | **24** |  |
| Informed Consent (a) | X |  |  |  |  |  |  |  |  |  |
| Inclusion/Exclusion Criteria | X | X |  |  |  |  |  |  |  |  |
| Pregnancy Test (WOCBP only) | X* |  |  |  |  |  |  |  |  |  |
| Hepatitis B, C, and HIV test (j) | X |  |  |  |  |  |  |  |  |  |
| Demography | X |  |  |  |  |  |  |  |  |  |
| Medical History | X |  |  |  |  |  |  |  |  |  |
| Prior Medication Collection | X |  |  |  |  |  |  |  |  |  |
| OPL Biopsy (b) | X |  |  |  |  |  |  |  | X |  |
| LOH assessment (c) | X |  |  |  |  |  |  |  | X |  |
| Assessment of Signs and Symptoms | X | X | X | X | X | X | X | X | X | X |
| PD-L1 expression (d) |  | X |  |  |  |  |  |  |  |  |
| Blood and Saliva Sample for Biomarker assessments (e) |  | X |  |  | X |  |  |  | X | X*** |
| Physical examination | X | X | X | X | X | X | X | X | X | X |
| Vital Signs and Oxygen Saturation (f) | X** | X | X | X | X | X | X | X | X | X |
| ECOG Performance Status | X | X | X | X | X | X | X | X | X | X |
| Full Blood Count, Blood Chemistry and Urine Test (g) | X** | X | X | X | X | X | X | X | X | X |
| ECG 12 leads | X** |  |  |  |  |  |  |  |  |  |
| Concomitant Medication Collection | X** | X | X | X | X | X | X | X | X | X |
| Narrow Band Imaging Assessment (h) |  | X* |  | X |  | X |  |  | X | X |
| AEs Assessment | X | X | X | X | X | X | X | X | X | X |
| Avelumab Administration (i) |  | X | X | X | X |  |  |  |  |  |

## Flow Chart/Time and Events Schedule

* Within 24 hours prior to the initial administration of study drug at baseline.

** Within 14 days prior to the initial administration of study drug at baseline.

***To be performed if OPL malignant transformation is confirmed.

- (a)  A signed, written informed consent form must be obtained from the patient prior to any study-specific procedures or assessments;
- (b)  Sufficient evaluable OPL tissue obtained before the start of study drug treatment and 6 months after the first drug administration (block or minimum of 10 slides containing a minimum of 100 evaluable OPL cells obtained from core biopsy, punch biopsy, excisional biopsy or surgical specimen; a fine needle biopsy or brushing are not sufficient).

If feasible, 6 months after the beginning of study drug treatment a complete resection of OPL will be performed.

- (c) LOH assessment. The following analysis will be performed to define LOH positivity: LOH at 3p14 and/or 9p21 plus at least at one additional chromosomal site (4q, 8p,11p,13q, or 17p) or patients with a prior oral cancer history and LOH at 3p14 and/or 9p21 (LOH defined according to EPOC trial).
- (d) PD-L1 expression by OPL cells will be measured by immunohistochemical analysis of formalin-fixed, paraffin-embedded blocks or slides.
- (e)  Blood (about 20 mL) and saliva sample (about 5 ml) at baseline, just after the 4^th^ administration of the drug, 6 months after start of treatment and at OPL malignant transformation. Detailed instructions of the obtaining, processing, labelling, handling, storage and shipment of specimens will be provided in a separate Work Instruction at the time of study initiation.
- (f)  Including blood pressure, heart rate, temperature, and oxygen saturation by pulse oximetry (at rest). Obtain vital signs at the screening visit and within 72 hours prior to the first dose.
- (g)  CBC w/differential, LFTs (ALT, AST, total bilirubin, alkaline phosphatase), BUN or serum urea level, creatinine, Ca, Mg, Na, K, Cl, LDH, CK, CRP, glucose, amylase, lipase, TSH with reflexive Free T4, Free T3, ACTH (only at visit 1) and urine chemical-physical examination. On-study local laboratory assessments should be done within 72 hours prior to each dose.
- (h)  Narrow band imaging (optional in selected centres)
- (i)  Avelumab monotherapy at 800 mg every 2 weeks as a 60-minute IV infusion on Day 1 of a treatment cycle every 2 weeks (14 days) up to a total of 4 administrations. Subjects may be dosed no less than 12 days between doses.
- (j)  Hepatitis B surface antigen (HBV sAg), and hepatitis C antibody (HCV Ab) or hepatitis C RNA (HCV RNA). Subjects who test positive for hepatitis C but test negative for ribonucleic acid are allowed to enrol.
